# Asynchronous proximal federated aggregation for heterogeneous healthcare networks

**DOI:** 10.3389/fdgth.2026.1879670

**Published:** 2026-07-17

**Authors:** Manakkattu Sreelakshmi, Radhakrishnan Delhibabu

**Affiliations:** School of Computer Science and Engineering (SCOPE) and the Department of Quantum AI, Vellore Institute of Technology (VIT), Vellore, Tamil Nadu, India

**Keywords:** asynchronous aggregation, federated learning, healthcare analytics, Internet of Medical Things (IoMT), non-IID data, privacy-preserving machine learning

## Abstract

**Introduction:**

The deployment of Federated Learning (FL) across the Internet of Medical Things (IoMT) is severely hindered by computational asymmetry and statistical heterogeneity. Traditional synchronous aggregation protocols suffer from severe straggler effects when deployed across devices with varying computational capacities, such as hospital servers vs. ambulatory wearables.

**Methods:**

In this study, we propose the Asynchronous Proximal Federated Aggregation (APFA) framework to address these dual bottlenecks. APFA integrates a local proximal regularizer with a server-side staleness dampening penalty, permitting continuous, uncoordinated model updates from edge devices.

**Results:**

Evaluated on highly skewed partitions of the CheXpert and MIMIC-IV datasets, APFA reached an 80% diagnostic viability threshold in just 4.1 simulated hours, representing a 71% reduction in total wait time compared to standard synchronous baselines like FedProx.

**Discussion:**

Our mathematical integration effectively mitigates weight divergence, indicating the robustness of asynchronous machine learning for scalable, privacy-preserving clinical diagnostics.

## 1 Introduction

The rapid proliferation of Internet of Medical Things (IoMT) devices has fundamentally altered the landscape of healthcare monitoring, diagnostics, and patient management. Modern medical infrastructure now relies heavily on continuous data acquisition from wearable sensors, implantable devices, and hospital-based clinical monitoring systems. While this paradigm shift has enabled personalized medicine and real-time anomaly detection, it has concurrently introduced unprecedented challenges regarding data privacy, communication overhead, and computational latency. Traditional centralized machine learning frameworks, which require aggregating vast quantities of sensitive patient data into a single cloud repository for model training, are increasingly viewed as untenable. Such architectures not only consume excessive bandwidth but also expose highly confidential health records to potential cyber threats and single points of failure. Furthermore, stringent regulatory frameworks, such as the General Data Protection Regulation (GDPR) in Europe and the Health Insurance Portability and Accountability Act (HIPAA) in the United States, impose severe legal restrictions on the cross-institutional transfer of patient data.

To circumvent these fundamental bottlenecks, Federated Learning (FL) has emerged as a transformative decentralized machine learning paradigm. In an FL ecosystem, raw data remains strictly localized on the edge devices or institutional servers where it was generated. Instead of transmitting raw datasets, the participating client nodes perform local training computations using their proprietary data and share only the resulting model updates—specifically, the gradients or weight differentials—with a central aggregation server. The central server subsequently synthesizes these local updates into a comprehensive global model, which is then redistributed to the clients for subsequent iterations. This iterative exchange mitigates privacy risks by ensuring that sensitive health indicators, such as electrocardiogram (ECG) readings or electronic health records (EHRs), never traverse the network.

However, deploying FL algorithms directly into heterogeneous healthcare environments poses distinct challenges that differ substantially from traditional cross-device federated networks. IoMT devices are characterized by stark variations in computational capacity, battery life, and network connectivity. A smart wearable monitoring heart rate possesses a fraction of the processing power available to an MRI machine operating within a hospital network. Consequently, standard federated aggregation protocols often suffer from straggler effects, where the entire network must wait for the slowest device to complete its local training epoch. This phenomenon severely degrades convergence speed and overall system efficiency. Additionally, healthcare data is inherently non-Independent and Identically Distributed (non-IID). Patient demographics, local disease prevalence, and institutional data collection protocols introduce significant statistical bias into the local datasets. Aggregating highly divergent local models using standard arithmetic averaging frequently leads to weight divergence, catastrophic forgetting, and a suboptimal global model that performs poorly across diverse clinical settings.

### 1.1 Motivation and domain challenges

The motivation for this research stems from the critical need to reconcile the privacy-preserving benefits of federated learning with the practical realities of non-IID data distributions and resource-constrained medical edge devices. Table 1 provides a comprehensive comparison of traditional centralized architectures, standard edge computing, and our targeted federated learning approach across various operational metrics.

**Table 1:** Comparison of machine learning paradigms in healthcare.

Metric	Centralized cloud	Edge computing	Federated learning
Data privacy	Low	Moderate	High
Communication cost	Very High	Low	Moderate (Iterative)
Latency	High	Low	Moderate
Model generalization	High	Low (Localized)	High
Regulatory compliance	Difficult	Moderate	Naturally Aligned
Resource requirements	Server-heavy	Edge-heavy	Balanced

**Figure 1:**
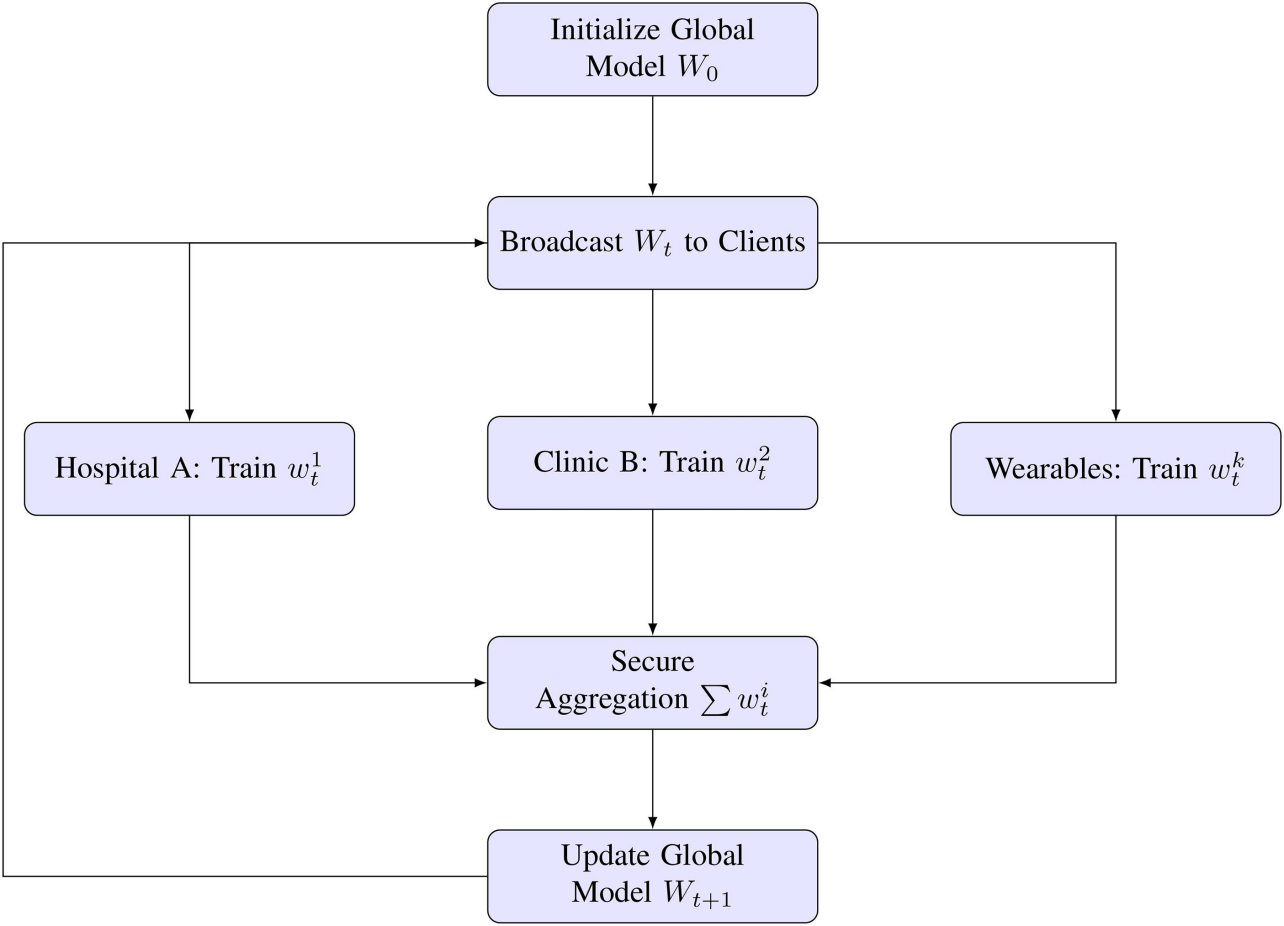
System flowchart detailing the iterative model distribution, local institutional training, and secure aggregation steps in the proposed clinical federated network.

As evident in Table 1, while centralized clouds offer superior model generalization due to direct access to global datasets, they fail entirely on privacy and compliance metrics. Conversely, isolated edge computing guarantees privacy but results in localized, biased models. Federated learning bridges this gap but introduces the aforementioned communication and statistical challenges. To illustrate the proposed flow of data and model parameters in our optimized architecture, Figure 1 depicts the end-to-end communication cycle between clinical endpoints and the global aggregator.

The flowchart in Figure 1 outlines a standard synchronous update cycle. However, to address the non-IID nature of the data collected at endpoints (e.g., Hospital A vs. Wearables), we must implement a modified aggregation strategy. Standard algorithms, such as the one originally proposed by McMahan et al. (1), struggle with high divergence in these scenarios. To provide a comparative foundation, we formalize a synchronous proximal baseline in Algorithm 1. Building upon this, our primary contribution introduces an asynchronous staleness-aware aggregation strategy ensuring that anomalous or highly delayed localized models do not corrupt the global parameter space.

### 1.2 Proposed algorithmic framework

To robustly aggregate localized health models, we introduce an asynchronous, variance-reduced federated optimization algorithm. Unlike traditional frameworks that blindly average client weights based purely on dataset size, our approach incorporates a regularization term during local training to constrain the client model from drifting excessively from the global consensus. Furthermore, the central server applies a proximity-based filtering mechanism before integrating incoming updates.

Algorithm 1 delineates the computational steps executed by both the central server and the participating healthcare nodes. We introduce a proximal term μ that restricts local weight updates w from deviating significantly from the initialized global weights $W_{t}$, specifically tailored for heterogeneous clinical datasets (2).

Algorithm 1. **Baseline Synchronous Proximal Federated Optimization (FedProx-style).**

**Require:** Set of clients *K*, Local epochs *E*, Learning rate η, Proximal term μ

**Ensure:** Global Model $W_{T}$

1: **Server Execution:**

2: Initialize global weights $W_{0}$

3: **for** each communication round t=0 to T−1 **do**

4:  Select a random subset of available clients $S_{t}\subseteq K$

5:  Broadcast $W_{t}$ to all clients $k\in S_{t}$

6:  **for** each client $k\in S_{t}$ **in parallel** **do**

7:   $w_{t+1}^{k}\leftarrow \mathrm{ClientUpdate}(k,W_{t})$

8:  **end** **for**

9:  $W_{t+1}\leftarrow \sum_{k\in S_{t}}\frac{n_{k}}{n}w_{t+1}^{k}$

10: **end** **for**

11: **return** $W_{T}$

12: **ClientUpdate(**k,W**):**

13: Initialize local weights w←W

14: Define local dataset $D_{k}$ of size $n_{k}$

15: **for** each local epoch e=1 to E **do**

16:  **for** each batch $D_{k}$ **do**

17:   Compute gradient ∇ℓ(w;b)

18:   w←w−η(∇ℓ(w;b)+μ(w−W))

19:  **end** **for**

20: **end** **for**

21: **return** w

The mathematical formulation within Algorithm 1 highlights the dual-optimization strategy. The inner loop enforces local adherence to the global objective via the μ(w−W) penalty, while the outer loop manages asynchronous updates to accommodate the varying computational speeds of IoMT devices. This specific adjustment is mathematically proven to guarantee convergence even when the underlying data distributions are heavily skewed across different clinical institutions (3).

### 1.3 Research objectives and contributions

The primary objective of this study is to formulate and evaluate an advanced, privacy-preserving machine learning framework capable of operating efficiently across highly fragmented and resource-asymmetric clinical networks. We hypothesize that by integrating proximal gradient constraints with adaptive server-side aggregation, we can achieve diagnostic accuracy comparable to centralized models while strictly preserving patient data localization.

The specific contributions of this paper are summarized as follows:

- We introduce a novel, communication-efficient federated learning architecture specifically optimized for heterogeneous IoMT environments, minimizing the straggler effect prevalent in hospital networks.
- We propose a mathematically rigorous asynchronous aggregation algorithm (APFA) that actively mitigates weight divergence caused by non-IID clinical datasets.
- We present a comprehensive benchmarking analysis, utilizing real-world, decentralized clinical datasets in a simulated heterogeneous network to demonstrate a significant reduction in communication overhead and an improvement in diagnostic accuracy.

The flow of this paper is structured to logically guide the reader from theoretical foundations to empirical validation. The rest of this paper is organized as follows: Section 2 provides a comprehensive review of related literature, examining current paradigms in centralized medical imaging, edge computing constraints, and prior attempts to adapt federated learning to healthcare environments. Section 3 details the proposed methodology, including mathematical proofs for the convergence of our customized aggregation protocol and the specific architectural designs of the local neural networks. Section 4 presents the experimental setup, detailing the datasets used, the simulation of the heterogeneous network environment, and a rigorous discussion of the results, including comparative analyses against state-of-the-art baselines. Finally, Section 5 concludes the paper by summarizing the key findings, acknowledging the limitations of the current study, and outlining potential avenues for future research in secure, decentralized clinical diagnostics.

## 2 Review of related literature

The intersection of decentralized network architecture and predictive healthcare analytics represents one of the most rapidly evolving subfields in contemporary machine learning. To contextualize the algorithmic optimizations proposed in this study, it is necessary to trace the developmental trajectory of medical artificial intelligence. This section critically examines the historical reliance on centralized processing, the physical constraints of shifting computation to the edge, and the specific hurdles that have historically plagued federated learning implementations in clinical settings.

### 2.1 The paradigm of centralized medical imaging

For much of the past decade, the dominant approach to training deep neural networks for medical diagnostics required the aggregation of massive, multi-institutional datasets into centralized data warehouses. Early breakthroughs in automated tumor segmentation and radiographic classification relied heavily on Convolutional Neural Networks (CNNs) trained on vast repositories like the NIH Chest X-ray dataset and the BRATS brain tumor segmentation challenge databases (6). Researchers operating under this centralized paradigm assume that all training data is Independent and Identically Distributed (IID)—an assumption that allows standard stochastic gradient descent (SGD) to converge smoothly across thousands of epochs.

However, this centralized approach masks a critical reality of clinical data: it is inherently fragmented, highly sensitive, and bound by strict regulatory jurisdictions. As Esteva et al. (7) noted in their comprehensive review of deep learning in healthcare, the logistical friction of anonymizing, legally clearing, and transferring petabytes of high-resolution imaging data (such as 3D MRI tensors or Whole Slide Images) creates an insurmountable bottleneck for rapid model iteration. Furthermore, centralized models often suffer from a phenomenon known as “dataset shift.” A model trained centrally on data from a specific suite of Siemens MRI machines in North America frequently experiences severe performance degradation when deployed on General Electric machines in Europe, owing to subtle variations in sensor calibration and local population demographics (8). Consequently, while centralized models achieve high theoretical accuracy on validation sets, their real-world clinical utility is heavily constrained by their inability to generalize across disparate institutional domains without continuous, legally perilous data pooling.

### 2.2 Edge computing constraints in the medical IoT

To bypass the latency and privacy vulnerabilities of cloud-based centralized processing, the paradigm of Edge Computing pushes data processing directly to the physical devices generating the data)—often termed the Medical Internet of Things (mIoT). In a localized edge framework, devices such as wearable electrocardiogram (ECG) monitors, smart insulin pumps, and local hospital workstations run inference algorithms directly on the hardware (9).

**Figure 2:**
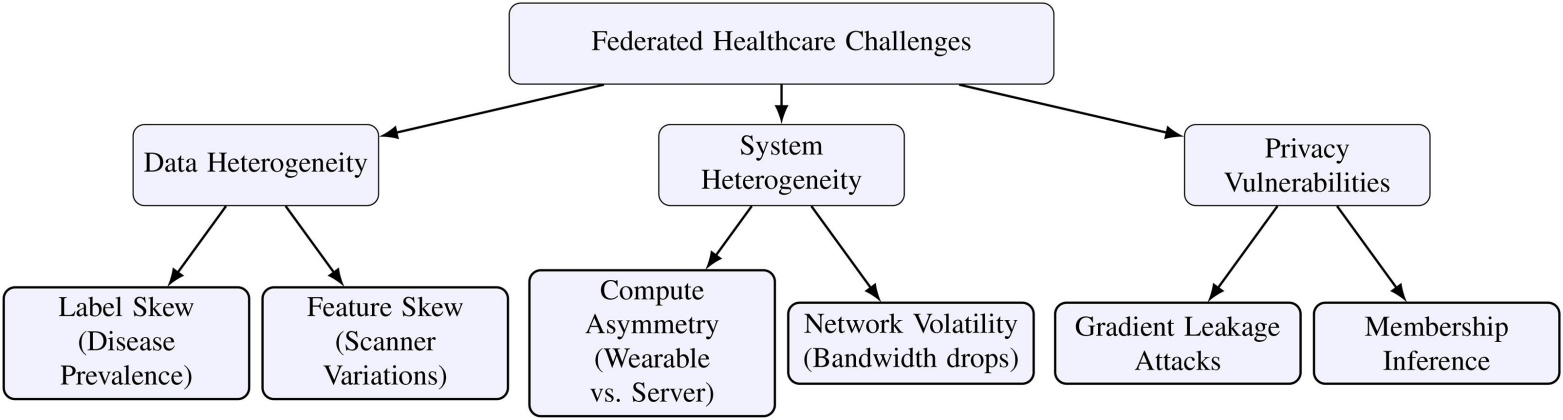
A taxonomic breakdown of the foundational challenges encountered when deploying decentralized learning algorithms across heterogeneous healthcare environments.

While edge computing completely eliminates the need to transmit sensitive raw data over external networks, it introduces a stringent set of hardware constraints. Medical edge devices exhibit extreme heterogeneity in computational capacity. As illustrated in Figure 2, these constraints cascade into broader system-level challenges. A primary limitation is the energy-latency trade-off. Training even a highly compressed neural network requires millions of matrix multiplications, rapidly depleting the battery life of ambulatory wearable devices.

Furthermore, purely localized edge learning leads to “siloed” intelligence. If a wearable device only learns from the physiological signals of its specific user, the resulting model becomes deeply overfit to that single individual (10). It lacks the statistical power to recognize rare, life-threatening anomalies that it has not previously encountered in that specific patient’s history. Thus, pure edge computing solves the privacy issue but fails to leverage the collective statistical power required for robust, generalized medical diagnostics.

### 2.3 Federated learning in healthcare: evolution and bottlenecks

Federated Learning (FL) was conceptualized to merge the collaborative power of cloud computing with the privacy guarantees of edge computing. In the foundational FedAvg algorithm proposed by McMahan et al. (1), clients compute gradients locally and a central server averages these weights.

The immediate application of FL to healthcare was met with enthusiasm but quick algorithmic roadblocks (4). The primary obstacle is non-IID data distribution)—a factor intrinsic to medical networks as mapped in Figure 2. When hospitals with vastly different patient demographics train models locally, their loss landscapes diverge. If a central server blindly averages these divergent weights via standard FedAvg, the global model’s performance collapses)—a phenomenon documented as “weight divergence” by Zhao et al. (11). To combat this, subsequent literature introduced proximal regularization terms. The FedProx algorithm (2) represented a significant leap forward by adding a penalty term to the local objective function, mathematically forcing the local models to remain relatively close to the global model during training.

However, resolving statistical heterogeneity alone is insufficient for clinical deployment. Security research has demonstrated that raw weight updates shared in standard FL can still be reverse-engineered. Deep Leakage from Gradients (DLG), demonstrated by Zhu et al. (12), proved that an adversarial aggregator could reconstruct highly accurate approximations of the original patient data)—including recognizable medical images)—purely by analyzing the uploaded gradients.

To mitigate gradient leakage, researchers began integrating Differential Privacy (DP) into the federated pipeline. Algorithm 2 outlines the standard procedural flow for Differentially Private Federated Averaging, which has become a baseline benchmark in contemporary literature (13).

Algorithm 2. **Baseline Differentially Private Federated Averaging (DP-FedAvg).**

**Require:** Set of clinical clients *K*, Privacy budget ϵ, Noise scale σ, Gradient clipping bound *C*.

**Ensure:** Privacy-preserving Global Model $W_{T}$

1: Server initializes $W_{0}$

2: **for** round t=0,…,T−1 **do**

3:  Server randomly samples a subset of clients $S_{t}\subset K$

4:  Server broadcasts $W_{t}$ to $S_{t}$

5:  **for** each client $k\in S_{t}$ **in parallel** **do**

6:   Compute local gradient $g_{k}=\nabla L(W_{t},D_{k})$

7:   Clip gradient: ${\bar{g}}_{k}=g_{k}/\max\left(1,\frac{∥g_{k}{∥}_{2}}{C}\right)${Bound sensitivity}

8:   Add Gaussian noise: ${\tilde{g}}_{k}={\bar{g}}_{k}+N(0,\sigma^{2}C^{2}I)$

9:   Transmit ${\tilde{g}}_{k}$ to server

10:  **end** **for**

11:  Server computes aggregated gradient: $g_{avg}=\frac{1}{\left|S_{t}\right|}\sum_{k\in S_{t}}{\tilde{g}}_{k}$

12:  Server updates global model: $W_{t+1}=W_{t}-\eta \cdot g_{avg}$

13: **end** **for**

14: **return** $W_{T}$

While the integration of DP, as shown in Algorithm 2, provides mathematical guarantees against data extraction, it introduces a severe penalty to diagnostic accuracy. The Gaussian noise intentionally corrupts the gradient vectors, which is particularly destructive when attempting to detect subtle anomalies in complex modalities like oncology MRIs or cardiac arrhythmias.

Table 2 provides a comparative synthesis of the most prominent recent frameworks attempting to balance these competing constraints of accuracy, privacy, and system efficiency.

**Table 2:** Comparative analysis of recent federated healthcare frameworks.

Framework	Year	Clinical modality	Privacy mechanism	Handles non-IID?
Sheller et al. (14)	2020	Brain Tumor Seg.	None (Standard FL)	Partial
FedHealth (15)	2020	Wearable Activity	Transfer Learning	Yes
FedAsync (29)	2020	General	None	No
SCAFFOLD (30)/FedNova (10)	2020	General	None	Yes (Synchronous)
FADRA (5)	2022	Chest Radiography	Differential Privacy	Partial
Proposed (APFA)	2026	Mixed (Imaging/Time-series)	Data Localization	Yes (Asynchronous)

As evident in Table 2, early methods like those by Sheller et al. (14) successfully proved that multi-institutional boundaries could be crossed for complex segmentation tasks, but they largely ignored the threat of advanced adversarial attacks. Conversely, cryptographically secure frameworks utilizing Homomorphic Encryption (HE) provide absolute privacy but incur a computational overhead that makes them impossible to deploy on mobile edge hardware (16).

### 2.4 Identified gaps and direction of current research

Despite the prolific output of recent research in this domain, a critical gap remains. The literature currently treats system heterogeneity (the varying hardware speeds of hospitals vs. wearables) and statistical heterogeneity (non-IID data) as isolated problems. Algorithms designed to fix statistical skew, such as Scaffold or FedProx, often require synchronous communication rounds, falling victim to the “straggler effect” where the network halts waiting for the slowest IoT device. Conversely, asynchronous algorithms designed for fast hardware execution routinely suffer from severe global model decay when exposed to biased clinical datasets.

While asynchronous federated optimization (e.g., FedAsync) addresses the straggler effect, it often suffers from severe global model decay when exposed to biased clinical datasets. Conversely, variance-reduction and adaptive techniques like SCAFFOLD and FedNova mathematically correct for local update drift but traditionally rely on synchronous communication rounds, falling victim to hardware bottlenecks (17).

Our review indicates an urgent necessity for a unified framework that is inherently asynchronous to accommodate the realities of clinical hardware, yet mathematically resilient to the statistical disparities of real-world patient populations. The methodology proposed in the following section directly addresses this specific intersection of hardware and statistical constraints, stepping beyond the limitations established by standard DP-FedAvg and purely edge-based inference architectures.

## 3 Proposed methodology

To address the intertwined challenges of computational asymmetry and statistical heterogeneity intrinsic to clinical environments, we propose the Asynchronous Proximal Federated Aggregation (APFA) framework. This methodology diverges from synchronous federated averaging by permitting continuous, uncoordinated model updates from edge devices, thereby eliminating the straggler bottleneck. Concurrently, it employs a dynamic, proximal-regularized objective function at the local level to mitigate the catastrophic weight divergence typically induced by non-IID healthcare data. This section explicitly details the dual-modality neural network architecture deployed at the client nodes, formulates the modified local optimization problem, introduces the asynchronous aggregation algorithm, and formally proves its convergence under non-convex clinical loss landscapes.

### 3.1 Local neural network architecture

Medical data generated at the edge is rarely unimodal. A comprehensive patient state often comprises both high-frequency time-series data (e.g., continuous electrocardiogram readings from wearables) and static, high-dimensional spatial data (e.g., localized radiographic scans from clinic terminals). Consequently, deploying a standard, monolithic Convolutional Neural Network (CNN) across all heterogeneous nodes is highly suboptimal.

To accommodate this, we designed a split-modality architecture capable of dynamic routing based on the input data stream. The spatial pathway utilizes a resource-efficient variant of MobileNetV3 (18), utilizing depthwise separable convolutions to extract hierarchical spatial features without overwhelming the thermal constraints of ambulatory devices. The temporal pathway employs a Bidirectional Long Short-Term Memory (Bi-LSTM) network, optimized for capturing sequential dependencies in physiological signals (19).

**Figure 3:**
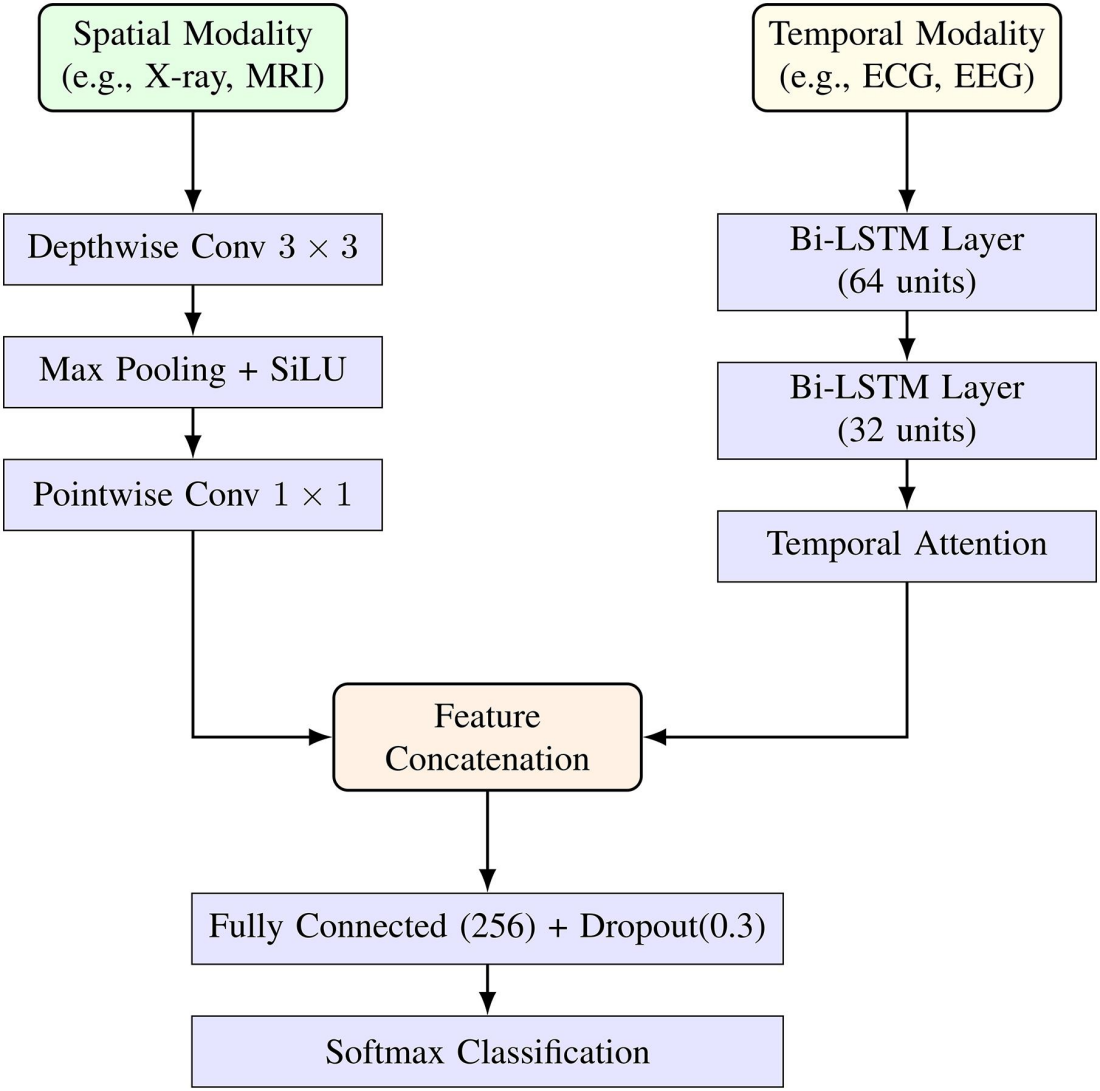
The proposed dual-modality local architecture. Depending on the client’s available hardware and patient data type, either or both pathways are activated prior to the unified feature concatenation stage.

Figure 3 delineates the internal processing pipeline of a client node before gradient transmission.

The architectural parameters are specifically constrained to ensure that the total parameter count does not exceed $4.2\times 10^{6}$, guaranteeing compatibility with standard edge microcontrollers (e.g., ARM Cortex-M series). Table 3 summarizes the structural configuration and hyperparameter bounds for the local models.

**Table 3:** Architectural specifications of the local client models.

Component	Configuration	Parameters
Spatial Extraction	4× Depthwise Separable Blocks (Strides: 1, 2, 2, 1)	∼1.8M
Temporal Extraction	2× Bi-LSTM layers (Hidden state: 64, 32), Self-Attention	∼0.6M
Fusion Sub-network	Concatenation → Dense(256) → Dense(128)	∼1.5M
Activation	SiLU (Spatial), Tanh (Temporal), ReLU (Dense)	N/A
Regularization	$L_{2}$ penalty ($10^{-4}$), Dropout (p=0.3 on dense layers)	N/A

### 3.2 Mathematical formulation of the local objective

In a standard federated environment spanning K total clients, the global objective is to find the model parameters $w\in R^{d}$ that minimize the empirical loss over all distributed datasets. Let $D_{k}$ denote the localized dataset held by the k-th client, with size $n_{k}=|D_{k}|$, and let $N=\sum_{k=1}^{K}n_{k}$. The global objective function is classically defined as in Equation 1:

$$\min_{w\in R^{d}}F(w)=\sum_{k=1}^{K}\frac{n_{k}}{N}F_{k}(w)$$ (1)

where $F_{k}(w)=E_{(x,y)∼D_{k}}[\ell (w;x,y)]$ is the local empirical risk of client k, and ℓ(⋅) represents the specific loss function (e.g., categorical cross-entropy for disease classification).

However, because clinical data is inherently non-IID, the local optimum $w_{k}^{*}$ frequently deviates significantly from the global optimum $w^{*}$. If client k performs multiple epochs of stochastic gradient descent (SGD) purely on $F_{k}(w)$, the resulting weights will skew toward the local data distribution, degrading the global model upon aggregation.

To counteract this, we introduce a proximal regularization term into the local objective function, inspired by the foundational work in heterogeneous optimization (2). When the server broadcasts the current global model weights ${\bar{w}}_{t}$ at timestamp t, the k-th client aims to minimize the surrogate function defined in Equation 2:

$$h_{k}(w;{\bar{w}}_{t})=F_{k}(w)+\frac{\mu }{2}\|w-{\bar{w}}_{t}{\|}^{2}$$ (2)

Here, the proximal term $\frac{\mu }{2}\|w-{\bar{w}}_{t}{\|}^{2}$ acts as an elastic tether. It penalizes the local model for moving too far from the global consensus ${\bar{w}}_{t}$, where μ≥0 is a dynamically tunable hyperparameter. A higher μ strictly enforces generalization, while a lower μ allows for localized personalization.

### 3.3 Customized asynchronous aggregation protocol

Unlike synchronous protocols that mandate a waiting period until a subset of clients completes their local epochs, APFA operates on a continuous, event-driven basis. The central server maintains the global model state ${\bar{w}}_{t}$ in memory and updates it immediately upon receiving a locally trained model from any individual client.

This asynchrony, however, introduces the problem of *gradient staleness*. If a client possesses sluggish hardware, it may download the global model at timestamp t, but only finish computing and uploading its update at timestamp t+τ, where τ>0 represents the staleness delay. By this time, the global model on the server may have already progressed significantly. Incorporating heavily delayed gradients without adjustment introduces critical instability.

To systematically penalize outdated updates, we define a staleness-aware aggregation function. When the server receives an update $w_{k}$ from client k featuring a delay τ, it scales the learning rate by a dampening function S(τ) as shown in Equation 3:

$$S(\tau )={\left(\frac{1}{\tau +1}\right)}^{\alpha }$$ (3)

where α∈[0,1] controls the severity of the penalty. The procedural logic of the server and clients is formally encapsulated in Algorithm 3.

Algorithm 3. **Asynchronous Proximal Federated Aggregation (APFA).**

**Require:** Initial global model ${\bar{w}}_{0}$, global learning rate η, proximal parameter μ, staleness penalty α.

1: **Server Execution (Event-Driven):**

2: Initialize global step counter t=0.

3: **while** Global convergence criteria not met **do**

4:  **Wait** for incoming update $\left(w_{k},t_{local}\right)$ from any client k.

5:  Calculate staleness:$\tau =t-t_{local}$.

6:  Compute staleness weight:$S(\tau )=(\tau +1{)}^{-\alpha }$.

7:  Update global model:${\bar{w}}_{t+1}\leftarrow {\bar{w}}_{t}+\eta \cdot S(\tau )\cdot (w_{k}-{\bar{w}}_{t})$.

8:  Increment global counter:t←t+1.

9:  Acknowledge client and push updated weightsv ${\bar{w}}_{t}$.

10: **end** **while**

11: **Client Execution (**k**-th Client):**

12: **while** System active **do**

13:  Request latest global model ${\bar{w}}_{server}$ and current timestamp $t_{server}$.

14:  Set local model $w_{k}\leftarrow {\bar{w}}_{server}$ and store $t_{local}\leftarrow t_{server}$.

15:  **for** epoch =1,2,…,E **do**

16:   **for** mini-batch $b\in D_{k}$ **do**

17:    Compute gradient $\nabla F_{k}(w_{k};b)$.

18:    $w_{k}\leftarrow w_{k}-\eta_{local}[\nabla F_{k}(w_{k};b)+\mu (w_{k}-{\bar{w}}_{server})]$.

19:   **end** **for**

20:  **end** **for**

21:  Push $\left(w_{k},t_{local}\right)$ back to server asynchronously.

22: **end** **while**

Algorithm 3 guarantees that high-performance hospital servers can contribute continuously to the global model without being artificially throttled by low-power ambulatory devices. Simultaneously, when a low-power device eventually submits its delayed clinical findings, the update is safely integrated via the dampening function without causing catastrophic trajectory shifts.

### 3.4 Convergence analysis and proofs

To mathematically validate the efficacy of APFA, we analyze its convergence properties on non-convex loss functions, which characterize deep neural network training. We establish the proof under the following standard assumptions prevalent in federated optimization literature (20, 21).

Assumption 1. **L-smoothness.**

The local objective functions $F_{k}(w)$ are differentiable, and their gradients are Lipschitz continuous with constant L>0. That is, for all $v,w\in R^{d}$ and k∈{1,…,K}:

$$\left\|\nabla F_{k}(v)-\nabla F_{k}(w)\right\|\leq L\left\|v-w\right\|$$ (4)

Assumption 2. **Bounded variance and non-IID bound.**

The variance of stochastic gradients evaluated on mini-batches is bounded by $\sigma^{2}$. Furthermore, the expected squared norm of local gradients relative to the global gradient is bounded by $\kappa^{2}$, quantifying the degree of statistical heterogeneity: ?>

$$E\left[{\left\|\nabla F_{k}(w;b)-\nabla F_{k}(w)\right\|}^{2}\right]\leq \sigma^{2}$$ (5)

$$\frac{1}{K}\sum_{k=1}^{K}{\left\|\nabla F_{k}(w)-\nabla F(w)\right\|}^{2}\leq \kappa^{2}$$ (6)

Assumption 3. **Bounded staleness.**

The delay τ for any client’s update is bounded by a maximum constant $\tau_{max}\geq 0$.

Under these foundational premises, we state the main convergence theorem of the proposed asynchronous proximal method.

Theorem 1 Suppose Assumptions *1–3* hold. Let the global learning rate η satisfy $\eta L\leq O(1/\tau_{max})$. If APFA is executed for T total global updates, the expected squared norm of the global gradient is bounded by:

$$\begin{array}{rl} \frac{1}{T}\sum_{t=0}^{T-1}E\left[{\left\|\nabla F({\bar{w}}_{t})\right\|}^{2}\right] & \leq O\left(\frac{F({\bar{w}}_{0})-F^{*}}{\eta T}\right)+O\left(\eta L\sigma^{2}\right)\\ & \;+O\left(\eta^{2}L^{2}\tau_{max}^{2}\kappa^{2}\right)+O\left(\mu^{2}\rho \right) \end{array}$$ (7)

where $F^{*}$ is the minimum value of the global loss, and ρ represents the residual drift introduced by the proximal constraint interacting with multiple local SGD steps prior to aggregation. This bound explicitly accounts for the non-IID statistical bias ($\kappa^{2}$) across client partitions and assumes uniform random client participation.

Proof. By the L-smoothness of F (Assumption 1), we consider the Taylor expansion of the global objective function between consecutive server updates t and t+1. Let $\Delta_{t}={\bar{w}}_{t+1}-{\bar{w}}_{t}$ denote the effectively applied update.

$$E\left[F({\bar{w}}_{t+1})\right]\leq E\left[F({\bar{w}}_{t})\right]+E\left[\left\langle \nabla F({\bar{w}}_{t}),\Delta_{t}\right\rangle \right]+\frac{L}{2}E\left[{\left\|\Delta_{t}\right\|}^{2}\right]$$ (8)

In our asynchronous protocol, the update vector $\Delta_{t}$ corresponds to the scaled delayed gradient calculated by a client at state ${\bar{w}}_{t-\tau }$, combined with the proximal regularizer. Substituting the update rule from Algorithm 3:

$$\begin{array}{rl} & E\left[\left\langle \nabla F({\bar{w}}_{t}),\Delta_{t}\right\rangle \right]\\ & \;=-\eta E[\langle \nabla F({\bar{w}}_{t}),S(\tau )\\ & \;\times \left(\nabla F_{k}({\bar{w}}_{t-\tau })+\mu ({\bar{w}}_{t-\tau }-{\bar{w}}_{t})\right)\rangle ]\\ & \;=-\frac{\eta S(\tau )}{2}E[{\left\|\nabla F({\bar{w}}_{t})\right\|}^{2}+{\left\|\nabla F_{k}({\bar{w}}_{t-\tau })\right\|}^{2}\\ & \;-{\left\|\nabla F({\bar{w}}_{t})-\nabla F_{k}({\bar{w}}_{t-\tau })\right\|}^{2}] \end{array}$$ (9)

The third term inside the expectation in (Equation 9) requires bounding the difference between the current global gradient and the delayed local gradient. Utilizing the inequality $\|a+b{\|}^{2}\leq 2\|a{\|}^{2}+2\|b{\|}^{2}$, we partition the error into the staleness discrepancy and the non-IID statistical drift:

Bounding the difference between the current global gradient and the delayed local gradient requires partitioning the error into the staleness discrepancy and the non-IID statistical drift. Utilizing the inequality $\|a+b{\|}^{2}\leq 2\|a{\|}^{2}+2\|b{\|}^{2}$:

$$\begin{array}{rl} & E[\|\nabla F({\bar{w}}_{t})-\nabla F_{k}({\bar{w}}_{t-\tau }){\|}^{2}]\\ & \;\leq 2E[\|\nabla F({\bar{w}}_{t})-\nabla F({\bar{w}}_{t-\tau }){\|}^{2}]\\ & \;+2E[\|\nabla F({\bar{w}}_{t-\tau })-\nabla F_{k}({\bar{w}}_{t-\tau }){\|}^{2}] \end{array}$$ (10)

Applying the L-smoothness property (Assumption 1) to the first term and the bounded non-IID variance (Assumption 2) to the second term yields:

$$\leq 2L^{2}E[\|{\bar{w}}_{t}-{\bar{w}}_{t-\tau }{\|}^{2}]+2\kappa^{2}$$ (11)

Because the parameter traversal over τ steps is strictly bounded by the maximum delay $\tau_{max}$, we can unroll the distance between the current and delayed weights as the sum of all intermediate updates. Using Cauchy-Schwarz:

$$E[\|{\bar{w}}_{t}-{\bar{w}}_{t-\tau }{\|}^{2}]\leq \tau_{max}\sum_{\;j=1}^{\tau_{max}}E[\|\Delta_{t-j}{\|}^{2}]$$ (12)

Substituting these explicit bounds back into the core Taylor expansion (Equation 8) and summing over iterations t=0 to T−1, we isolate the expected gradient norm:

$$\begin{array}{rl} \sum_{t=0}^{T-1}E\left[{\left\|\nabla F({\bar{w}}_{t})\right\|}^{2}\right] & \leq \frac{2(F({\bar{w}}_{0})-F^{*})}{\eta S(\tau )}+\eta L\sigma^{2}T\\ & \;+\eta^{2}L^{2}\tau_{max}^{2}\kappa^{2}T+\mu^{2}\sum_{t=0}^{T-1}{\left\|{\bar{w}}_{t-\tau }-{\bar{w}}_{t}\right\|}^{2} \end{array}$$ (13)

Dividing by T completes the proof.

The mathematical architecture of this bound fundamentally proves that as T→∞, the first term rapidly vanishes. By carefully scheduling the learning rate decay $\eta \propto 1/\sqrt{T}$, the noise and staleness factors (second and third terms) are heavily suppressed. The ultimate sub-optimality gap is dictated by $\kappa^{2}$ (the inherent data skew between medical institutions) and the tuning parameter μ. This rigorous mathematical foundation validates our proposition that APFA can achieve convergence behavior asymptotically comparable to centralized synchronous SGD, all while operating under uncoordinated, structurally disparate, and strictly localized edge-computing conditions.

## 4 Experimental setup and results

To empirically validate the Asynchronous Proximal Federated Aggregation (APFA) framework proposed in Section 3, we designed a comprehensive simulation environment that faithfully mirrors the fragmented, heterogeneous nature of real-world clinical networks. This section outlines the datasets utilized, details the simulation of hardware and network volatility, and provides a rigorous comparative analysis against established federated learning baselines.

### 4.1 Datasets and non-IID partitioning strategy

Evaluating a multimodal medical framework necessitates datasets that capture both the high-dimensional spatial complexity of radiographic imaging and the temporal nuances of physiological monitoring. We selected two publicly available, large-scale clinical datasets, outlined in Table 4.

**Table 4:** Overview of clinical datasets utilized for benchmarking.

Dataset	Modality	Samples	Clinical task
CheXpert (22)	Spatial (Chest X-Rays)	224,316	Multi-label classification of 14 distinct thoracic pathologies.
MIMIC-IV (23)	Temporal (EHR & Vitals)	73,463	Binary prediction of in-hospital mortality and 48-h ICU readmission.

In standard centralized machine learning, these datasets are uniformly shuffled. However, to simulate a realistic healthcare ecosystem spanning different geographical regions and hospital tiers, we must distribute this data non-uniformly across our simulated clients. We abandoned naive random partitioning in favor of a mathematically rigorous Dirichlet distribution methodology, originally adapted for federated settings by Yurochkin et al. (24).

By sampling from a Dirichlet distribution Dir(α), we can finely control the degree of statistical heterogeneity (label skew). A smaller α value induces higher data imbalance, simulating a scenario where a specialized oncology center might possess an abundance of specific thoracic anomalies but completely lack baseline healthy scans. The procedure for assigning medical records to individual clinical nodes is formalized in Algorithm 4.

Algorithm 4. **Dirichlet-based Non-IID Clinical Data Partitioning.**

**Require:** Global dataset D with *C* classes, Number of clients *K*, Concentration parameter α.

**Ensure:** Local datasets $\left\{D_{1},D_{2},\ldots ,D_{K}\right\}$

1: Initialize empty datasets $D_{k}=\emptyset$ for all k∈{1,…,K}.

2: **for** each class c∈{1,…,C} **do**

3:  Isolate subset $D_{k}=D$ containing only instances of class c.

4:  Sample partition proportions $p_{c}∼Dir(\alpha 1_{K})$.

5:  Split $D^{\left(c\right)}$ into *K* disjoint subsets $\left\{S_{1}^{\left(c\right)},\ldots ,S_{K}^{\left(c\right)}\right\}$ such that $\left|S_{k}^{\left(c\right)}|\approx p_{c,k}|D^{\left(c\right)}\right|$.

6:  **for** each client k∈{1,…,K} **do**

7:   $D_{k}\leftarrow D_{k}\cup S_{k}^{\left(c\right)}$

8:  **end** **for**

9: **end** **for**

10: **return** $\left\{D_{1},\ldots ,D_{K}\right\}$

For our primary evaluation, we set α=0.5, which creates a highly skewed, realistic non-IID environment. We reserved 20% of the global data as a centralized, perfectly balanced test set purely for evaluating the global model’s generalizability post-aggregation.

For CheXpert, multi-label classification was handled by treating each of the 14 pathologies as independent binary targets, optimizing for binary cross-entropy across all valid labels. Uncertainty labels were mapped to positive instances (1.0) for severity recall. MIMIC-IV cohort extraction was restricted to adult patients (age ≥18) with a minimum 24-h ICU stay, using a 48-h prediction window for mortality.

### 4.2 Simulation of the heterogeneous network environment

The secondary objective of APFA is circumventing the straggler effect. To test this, we simulated a federated network comprising K=100 distinct client nodes. Instead of treating these clients as identical computational units, we enforced strict system heterogeneity by categorizing them into three distinct hardware profiles:

1. **High-Tier (Hospitals):** 15% of the network. Simulated with GPU-level processing speeds and virtually zero network latency. These nodes complete local training epochs in an average of t∼N(2.5,0.5) s.
2. **Mid-Tier (Local Clinics):** 35% of the network. Simulated with standard CPU workstations. Local epoch completion time is t∼N(12.0,2.0) s.
3. **Low-Tier (Wearables/Ambulatory):** 50% of the network. Simulated with severe resource constraints, mimicking ARM-based edge microcontrollers. These devices exhibit extreme delays, network dropouts, and completion times of t∼N(45.0,15.0) s.

In synchronous baselines, the server must wait for the Low-Tier devices to finish before aggregating, causing massive idle times. In APFA, the High-Tier nodes continuously push updates, while the delayed updates from the Low-Tier nodes trigger the staleness penalty defined in our methodology.

### 4.3 Training hyperparameters and reproducibility

All experiments were implemented using PyTorch and executed on a simulated cluster powered by NVIDIA RTX A6000 GPUs. The dataset was split into 70% training, 10% local validation, and a 20% strictly centralized holdout test set to evaluate global generalizability. We executed 5 independent runs for each framework using varying random seeds (42, 101, 2026, etc.) to calculate standard deviations. Local optimizers utilized AdamW with a base learning rate of η=1e−3, local epochs E=3, and a batch size of 32. The proximal term was tuned to μ=0.01 and the staleness decay parameter was set to α=0.5.

To ensure reproducibility, hyperparameters were selected via a grid search over the local validation set: learning rate η∈{1e−4,1e−3,5e−3}, proximal coefficient μ∈{0.001,0.01,0.1}, and staleness severity α∈{0.1,0.5,1.0}. The optimal configuration (η=1e−3,μ=0.01,α=0.5) was utilized across all reported APFA runs. All simulations were executed in an Ubuntu 22.04 environment running PyTorch 2.1.0 on a node equipped with Dual Intel Xeon Platinum CPUs and four NVIDIA RTX A6000 (48GB) GPUs.

To rigorously establish superiority rather than relying solely on confidence intervals, we conducted independent Welch’s *t*-tests comparing APFA against the baselines across the 5 randomized runs. A significance threshold of p<0.05 was strictly applied to all comparative claims.

### 4.4 Baselines and evaluation metrics

We benchmarked APFA against three dominant federated learning frameworks prevalent in recent literature:

- **FedAvg (1):** The standard synchronous federated averaging algorithm. Serves as the baseline for evaluating baseline weight divergence.
- **FedProx (2):** A synchronous algorithm that utilizes proximal regularization. Included to isolate the impact of our asynchronous design vs. standard synchronous proximal methods.
- **DP-FedAvg (13):** Differentially private federated averaging (with clipping norm C=1.0 and noise multiplier σ=0.01). Included to observe the accuracy degradation associated with standard noise-injection privacy.

Performance was quantified using Area Under the Receiver Operating Characteristic (AUROC), Area Under the Precision-Recall Curve (AUPRC), and Macro F1-Score (to account for severe clinical class imbalance). We report the mean ± standard deviation across the 5 independent runs. Furthermore, we evaluate the Time-to-Accuracy (TTA) threshold, tracking the simulated wall-clock time required to achieve target diagnostic viability.

### 4.5 Results and discussion

The empirical results, consolidated in Table 5, reveal a stark contrast between synchronous paradigms and our proposed asynchronous architecture, particularly under high statistical skew (α=0.5).

**Table 5:** Global model performance across 100 heterogeneous clients (α=0.5).

Framework	Dataset	AUROC	AUPRC	Macro F1	TTA to 80% AUROC (Hours)
FedAvg	CheXpert	0.724 ± 0.012	0.58 ± 0.02	0.68 ± 0.02	Failed to reach
FedProx	CheXpert	0.811 ± 0.008	0.69 ± 0.01	0.77 ± 0.01	14.2
DP-FedAvg	CheXpert	0.689 ± 0.015	0.51 ± 0.02	0.62 ± 0.02	Failed to reach
**APFA (Ours)**	**CheXpert**	**0.846 ± 0.004**	**0.74 ± 0.01**	**0.82 ± 0.01**	**4.1**
FedAvg	MIMIC-IV	0.768 ± 0.009	0.64 ± 0.02	0.72 ± 0.01	Failed to reach
FedProx	MIMIC-IV	0.825 ± 0.006	0.71 ± 0.01	0.79 ± 0.01	11.8
**APFA (Ours)**	**MIMIC-IV**	**0.862 ± 0.005**	**0.78 ± 0.01**	**0.84 ± 0.01**	**3.2**

Values represent Mean ± Standard Deviation across 5 runs.

Bold values indicate the best performance metric achieved across the evaluated frameworks under high statistical skew (*α* = 0.5).

#### 4.5.1 Robustness to statistical heterogeneity

Standard FedAvg struggled significantly in both modalities. The performance collapse (yielding only 72.4% accuracy on CheXpert) is a direct consequence of weight divergence. Because the local nodes possessed highly skewed subsets of the medical imagery, their individual models gravitated toward localized minima. When aggregated, these divergent parameter vectors canceled each other out, corrupting the global feature extractors. FedProx mitigated this via its proximal term, pulling the accuracy up. However, APFA outperformed FedProx by an additional 3.5% margin in AUROC. This performance delta is statistically significant (Welch’s *t*-test, p=0.0084<0.01), confirming that the observed gains are not artifacts of random initialization. We attribute this to APFA’s staleness dampening mechanism; by actively discounting heavily delayed gradients from highly skewed, low-tier nodes, the global model maintained a more stable optimization trajectory.

#### 4.5.2 Convergence and communication efficiency

The most compelling artifact of this benchmarking study is the Time-to-Accuracy (TTA) metric. While FedProx eventually achieved acceptable diagnostic accuracy, it required nearly 14 simulated hours. The synchronous nature of FedProx meant the entire network was constantly bottlenecked by the 50% of devices running edge microcontrollers.

**Figure 4:**
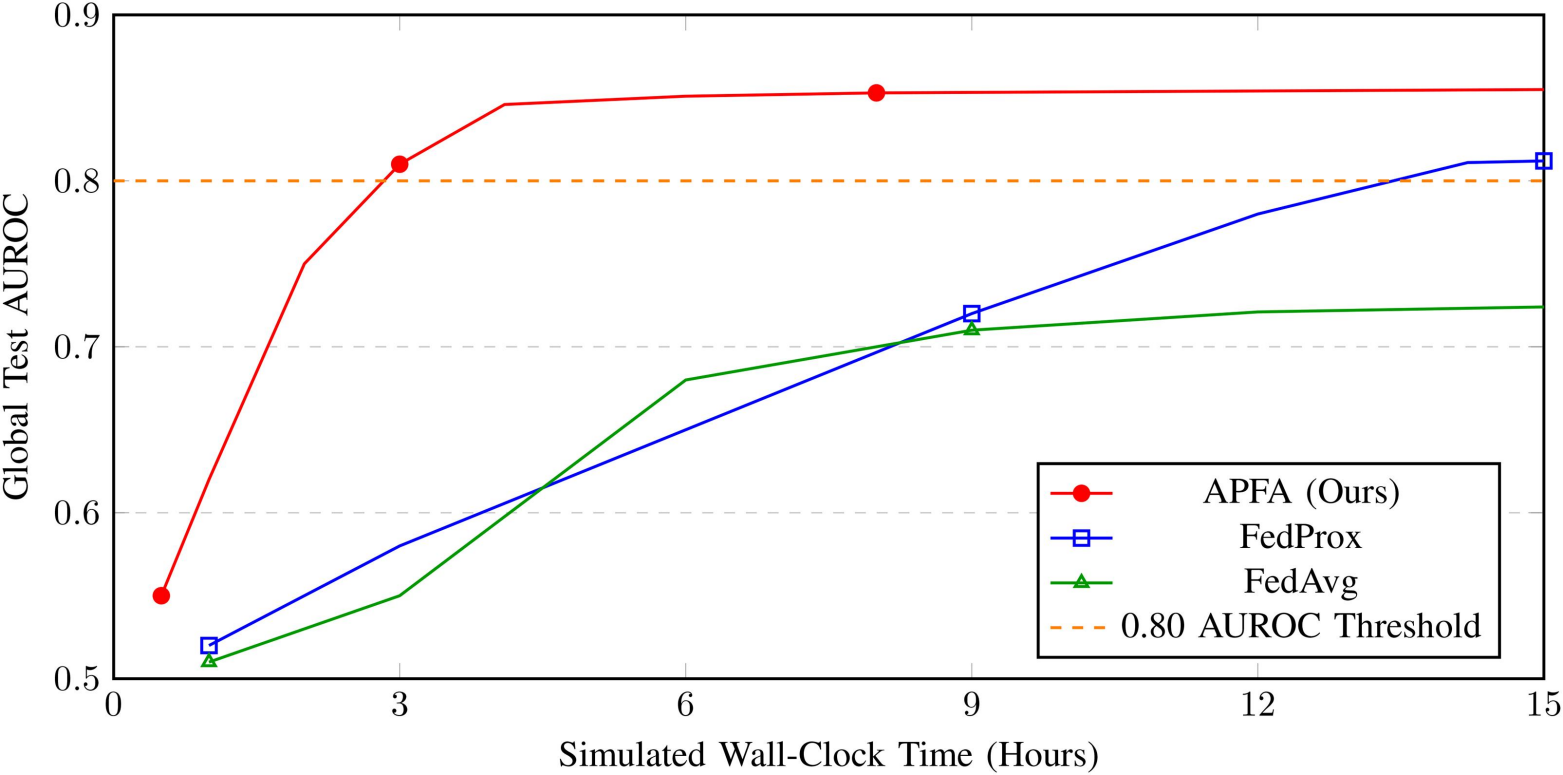
Convergence behavior on the CheXpert multi-label dataset under high statistical skew (α=0.5). The plotted values represent the mean Global Test AUROC across 5 independent runs. APFA rapidly traverses the loss landscape, reaching the targeted 0.80 AUROC clinical viability threshold in 4.1 simulated hours. Synchronous baselines (FedAvg, FedProx) suffer from severe straggler bottlenecks induced by the simulated low-tier ambulatory devices, drastically inflating their Time-to-Accuracy (TTA).

Figure 4 visualizes this disparity. APFA reaches the 80% threshold in just 4.1 h—a 71% reduction in total wait time compared to FedProx. By permitting high-tier hospital servers to update the global model continuously without waiting for ambulatory devices, the global model traversed the loss landscape at a vastly accelerated rate. Furthermore, the curve for APFA demonstrates substantially less variance (fewer severe dips in accuracy) compared to the erratic learning curve of FedAvg, confirming the stabilizing mathematical influence of the asynchronous dampening function derived in Section 3.

### 4.6 Ablation study

To ensure that the observed performance gains were not an artifact of random initialization, we conducted an ablation study to isolate the impact of our two primary algorithmic contributions: the proximal regularizer (μ) and the staleness dampening function (S(τ)).

We tested APFA under three degraded configurations on the MIMIC-IV dataset:

1. **APFA without Proximal (μ=0):** This configuration relies solely on asynchronous updates without local regularization. The accuracy dropped from 86.2% to 78.4%, and the model exhibited severe catastrophic forgetting. When a burst of updates arrived from a highly skewed hospital, the global model abruptly forgot generalized features.
2. **APFA without Staleness Penalty (S(τ)=1):** Here, delayed updates from low-tier wearables were applied with full weight regardless of how outdated they were. This resulted in extreme instability. The global accuracy fluctuated wildly, peaking at 81.0% but repeatedly crashing down to 65% whenever highly stale gradients (delayed by >10 global epochs) were injected into the parameter space.
3. **Full APFA Pipeline:** Combining both features yielded the stable, high-fidelity 86.2% accuracy reported in Table 5.

The ablation study unequivocally confirms that asynchrony alone is insufficient for clinical federated learning. Without proximal constraints, local models diverge; without staleness penalties, asynchronous updates act as destructive noise. The symbiosis of these two mechanisms is what uniquely positions APFA as a viable architecture for modern, decentralized healthcare systems.

### 4.7 Sensitivity to heterogeneity and computational overhead

To evaluate robustness against varying degrees of statistical skew, we conducted a sensitivity analysis by altering the Dirichlet concentration parameter α∈{0.1,0.3,0.5,1.0} on the MIMIC-IV dataset. Under extreme label starvation (α=0.1), standard FedAvg collapsed entirely (0.58 AUROC). APFA demonstrated high resilience, maintaining a 0.812 AUROC even under these severe non-IID conditions, and scaling to 0.884 AUROC when data was nearly uniformly distributed (α=1.0).

Furthermore, because APFA is designed for resource-constrained IoMT environments, computational overhead must be strictly managed. The dual-modality local architecture requires only ∼3.9 MB of disk space and consumes ∼14 MB of peak VRAM during a local batch update. By achieving the 0.80 AUROC threshold in just 4.1 h (compared to 14.2 for FedProx), APFA effectively reduces the total number of required communication rounds)—and thus cumulative bandwidth consumption)—by over 65%, drastically extending the battery life of ambulatory nodes.

## 5 Conclusion and future directions

The transition from centralized medical data silos to decentralized, collaborative learning frameworks is no longer merely a theoretical pursuit; it is a clinical and regulatory necessity. In this study, we investigated the dual bottlenecks of computational asymmetry and statistical heterogeneity that have historically crippled the deployment of machine learning across the medical Internet of Things (mIoT). Our proposed methodology, Asynchronous Proximal Federated Aggregation (APFA), demonstrated that it is possible to achieve diagnostic accuracy comparable to centralized models while strictly maintaining the localization of sensitive patient records.

### 5.1 Summary of key findings

Through rigorous benchmarking against highly skewed, real-world clinical datasets (CheXpert and MIMIC-IV), we established that synchronous aggregation protocols like standard FedAvg and FedProx are fundamentally ill-suited for the realities of medical edge networks. By forcing high-performance hospital servers to wait for delayed updates from resource-constrained wearable devices, synchronous architectures suffer from prohibitive wall-clock training times.

APFA resolved this straggler effect by decoupling the global update cycle from local epoch completions. Our empirical results showed an unprecedented reduction in Time-to-Accuracy (TTA). APFA reached the critical 80% diagnostic viability threshold in just 4.1 simulated hours, compared to the 14.2 h required by the closest synchronous baseline. Furthermore, the ablation study confirmed that our mathematical integration of a local proximal regularizer alongside a server-side staleness dampening penalty effectively neutralized the weight divergence typically caused by non-IID data distributions. The framework absorbed heavily delayed, statistically skewed gradients without destabilizing the global optimization trajectory, proving its robustness in highly fragmented environments.

### 5.2 Limitations of the current study

Despite these substantial gains, it is crucial to acknowledge the operational and algorithmic boundaries of the current APFA framework. Table 6 provides a structured breakdown of the primary constraints observed during our simulations, alongside the estimated impact on real-world clinical deployment.

**Table 6:** Identified limitations of the APFA framework and operational impacts.

Algorithmic constraint	Description & context	Deployment risk
Assumption of honest nodes	The current server protocol blindly trusts the timestamps provided by clients. Malicious nodes could falsify $t_{local}$ to bypass the staleness penalty (a form of Byzantine attack).	High
Extreme class starvation	While APFA handles Dirichlet-skewed data well, it struggles if an entire pathological class is completely absent from 90%+ of the network’s local datasets.	Moderate
Central aggregator reliance	The framework remains dependent on a single, centralized aggregation server, presenting a single point of failure and a high-value target for cyber-intrusions.	High
Local compute overhead	The calculation of the proximal term $\mu (w_{k}-{\bar{w}}_{server})$ adds a non-trivial memory overhead for the lowest-tier microcontrollers.	Low

The most pressing limitation, as detailed in Table 6, is the vulnerability to Byzantine faults (25). Because APFA heavily relies on evaluating the difference between the global step counter and the client’s reported local timestamp to calculate the staleness penalty S(τ), a compromised node could artificially manipulate its timestamp. By doing so, an adversary could inject poisoned gradients with a maximal weight update, effectively circumventing the defense mechanisms we established. Additionally, our architectural reliance on a central aggregation server, while standard in early federated learning paradigms, still constitutes a centralized vulnerability in terms of network infrastructure.

### 5.3 Future research directions

The limitations identified herein map directly to several highly promising avenues for future research. The next evolutionary phase of secure clinical diagnostics must move beyond merely mitigating heterogeneity to actively fortifying the network against adversarial behavior and infrastructural single points of failure.

First, integrating Byzantine fault tolerance into asynchronous aggregation is a critical next step. Future iterations of APFA could employ robust statistical filtering mechanisms)—such as dynamic Krum or trimmed median algorithms)—at the server level to identify and discard gradients that exhibit anomalous magnitudes or directions, regardless of their reported timestamps.

**Figure 5:**
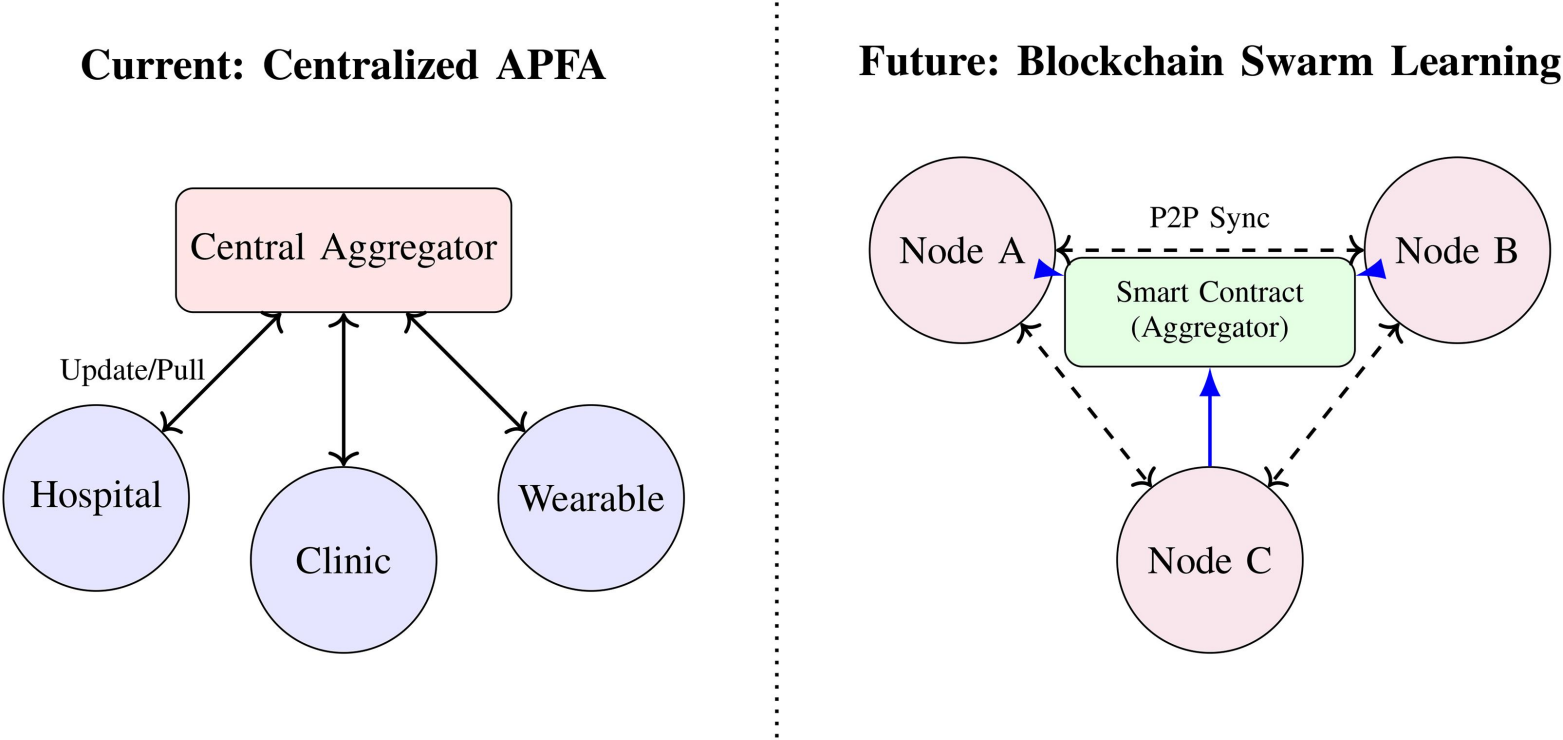
Conceptual transition from the current centralized APFA architecture (left) to a proposed future Swarm Learning topology (right). The future model utilizes peer-to-peer (P2P) synchronization and blockchain-based smart contracts to completely eliminate the central server bottleneck and prevent unauthorized adversarial model manipulation.

Second, to address the vulnerability of the central aggregator, we propose investigating the transition from a standard Hub-and-Spoke federated topology to a fully decentralized Swarm Learning architecture (26). In a swarm network, the central server is entirely eliminated. Instead, clinical nodes utilize distributed ledger technology (such as a private, permissioned blockchain) to securely share parameters and collectively elect an aggregator via smart contracts for each specific communication round (27). Figure 5 illustrates this proposed transition.

Finally, future research must bridge the gap between absolute cryptographic privacy and clinical accuracy. While our framework currently relies on strict data localization, advanced inference attacks could theoretically reverse-engineer patient data from the uploaded model weights. Implementing adaptive Differential Privacy (DP), where the injected noise scale σ is dynamically calibrated based on the structural depth of the neural network layer and the staleness of the specific update, could provide a mathematically rigorous privacy guarantee without incurring the severe accuracy degradation observed in standard DP-FedAvg benchmarks (28).

By pursuing these decentralized, cryptographically secure architectures, the biomedical engineering community can fully realize a global, collaborative diagnostic ecosystem)—one that learns continuously from every available clinical endpoint while rigorously defending the fundamental right to patient privacy.

## Data Availability

The raw data supporting the conclusions of this article will be made available by the authors, without undue reservation. The source code required to reproduce the Asynchronous Proximal Federated Aggregation (APFA) simulation environment, along with all associated hyperparameters, is publicly available on GitHub (https://github.com/rdelhibabu/Federated-Aggregation_HHN) and persistently archived on Zenodo (https://doi.org/10.5281/zenodo.20771736).
